# A True Case of a Broken Heart With Takotsubo Cardiomyopathy

**DOI:** 10.7759/cureus.21337

**Published:** 2022-01-17

**Authors:** Michelle Wallen, Graham E Kupsaw, Latha Ganti

**Affiliations:** 1 Emergency Medicine, University of Central Florida College of Medicine, Orlando, USA; 2 Neuroscience, Brown University, Providence, USA; 3 Emergency Medicine, Envision Physician Services, Plantation, USA; 4 Emergency Medicine, HCA Healthcare Graduate Medical Education Consortium Emergency Medicine Residency Program of Greater Orlando, Orlando, USA

**Keywords:** left ventricle, ventricular dysfunction, heart ventricles, cardiomyopathies, takotsubo cardiomyopathy

## Abstract

Takotsubo cardiomyopathy is characterized by transient regional systolic dysfunction of the left ventricle (LV) and mimics myocardial infarction. The LV displays a systolic apical ballooning appearance in this particular cardiomyopathy. This case demonstrated a patient with true stress-induced cardiomyopathy or “broken heart,” presenting to the emergency room with chest pain mimicking a non-ST elevation myocardial infarction.

## Introduction

Takotsubo (stress) cardiomyopathy presents as the characteristic ballooning of the left ventricular apex [1]. “Takotsubo” is a Japanese word that means “octopus pot”. The disease is characterized by transient systolic and diastolic left ventricular dysfunction with a variety of different wall-motion abnormalities [1,2]. Takotsubo cardiomyopathy typically affects elderly women, and it is a reversible cardiac syndrome.

The diagnosis is based on the exclusion of coronary disease. Most patients present with similar symptoms, which include physical or emotional stress, substernal chest discomfort and/or dyspnea, electrocardiogram (EKG) changes (ST elevation, T wave inversions), depressed left ventricular function on EKG, mild increase in cardiac enzymes, and no significant atherosclerotic coronary artery stenosis on coronary angiogram [3-5]. Because of the features of the disease, it is difficult initially to rule out acute coronary syndrome.

A case series showed that Takotsubo was diagnosed in patients after they had experienced severe emotional stress due to financial instability, severe occupation-related emotional stress, and emotional stress after the death of a relative [5]. These patients did have underlying risk factors, including hypertension and hyperlipidemia, and they presented with symptoms such as chest pain, dyspnea, and nausea. They either had ST elevation or marked T wave inversions on their EKGs. Each had mildly elevated cardiac enzymes, which painted the picture of acute coronary syndrome. On emergency cardiac catheterization, there was no obstructive coronary disease. Each, however, did show systolic apical ballooning with mild basal hyper-contraction on left ventriculography, diagnostic of Takotsubo [4,5].

One study showed that Takotsubo cardiomyopathy may be more common (5.7%) than previously reported (1 to 2.2%) in postmenopausal women presenting with the classical presentation of acute coronary syndrome [6]. Because of possible under-diagnosis, the study recommended that Takotsubo should be considered in all hospitalized patients with unexplained heart failure or left ventricular dysfunction [6].

## Case presentation

An 81-year-old female with a past medical history of hypertension and hyperlipidemia presented to the emergency room for chest pain. The patient admitted that the chest pain started 5 hours before arrival. At that time, she had received a phone call from a family member informing her that her youngest brother had died during elective surgery. She stated the pain started shortly after the call. The pain was substernal in origin with no radiation of the pain. She described the pain as waxing and waning, but would not resolve. The patient was stoic but admitted that her pain was a 10/10. She denied any shortness of breath, nausea or vomiting, or any recent illnesses. The patient had no known cardiac disease and denied having a stress test or echocardiogram in the past.

The patient’s vitals consisted of a pulse oximetry of 99%, a temperature of 37.1 °C, blood pressure of 162/88 mmHg, a heart rate of 99 beats per minute, and a respiratory rate of 18 breaths per minute. Her EKG showed normal sinus rhythm at 86 beats per minute with a 1st-degree atrioventrocular block, anterior infarct age undetermined, and left axis deviation with ST elevation in the single lead of V2 and ST depressions in I and aVL. On the exam, the patient was awake and alert. She was in no acute distress but did appear uncomfortable. She had no other significant findings on a physical exam. Labs were significant for a troponin of 11.62 ng/mL and second troponin of 12.80 ng/mL, a creatinine of 1.4 mg/dL, and a white blood cell count of 14.5K/mm^3^ (Table 1).

**Table 1:** Patient's laboratory values

Test	Value	Normal Range and Units
Sodium	136	135 - 145 mmol/L
Potassium	4.0	3.5 - 5.3 mmol/L
Chloride	101	99 - 111 mmol/L
Carbon Dioxide	24	21 - 32 mmol/L
Anion Gap	13.2	
Blood Urea Nitrogen	38 (High)	7 - 22 mg/dL
Creatinine	1.4 (High)	0.6 - 1.3 mg/dL
Estimated GFR	52	> 60
Glucose	102	70 - 110 mg/dL
Calcium	9.4	8.4 - 10.2 mg/dL
Troponin	11.69 (High)	0.0-0.4 ng/mL
3 hour Troponin	12.80 (High)	0.0-0.4 ng/mL
White blood cell count	14.5 (High)	4.1 - 9.3 K/mm3
Red Blood cell count	4.44	3.66 - 5.56 M/mm3
Hemoglobin	14.2	13.8 - 17.2 gm/dL
Hematocrit	42.6	40.6 - 51.8 %
Platelet count	233	150 - 450 K/mm3

With her abnormal EKG and significantly elevated troponin, cardiology was consulted. The patient had already received aspirin, a nitroglycerin drip, and 2 mg of morphine. Heparin bolus and drip were initiated, and the patient was admitted to the cardiovascular ICU for further evaluation, echocardiogram, and cardiac catheterization.

Echocardiogram revealed severely reduced left ventricular systolic function. There were regional wall motion abnormalities and severe mid-distal anterior and apical hypokinesis. Cardiac catheterization was performed, which showed mild non-obstructive epicardial coronary artery disease, apical ballooning with severe LV systolic dysfunction, and an ejection fraction of 25%.

## Discussion

**Figure 1:**
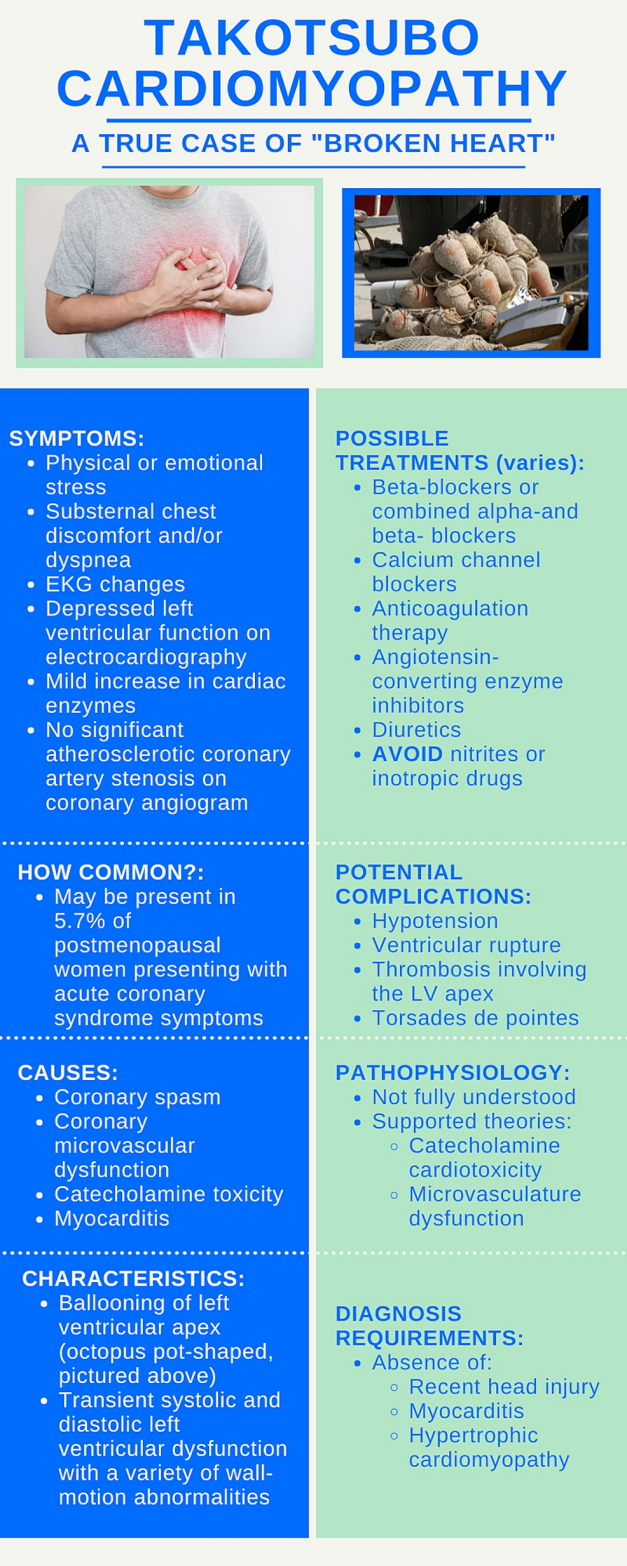
Infographic summarizing takotsubo cardiomyopathy. Designed by Graham Kupsaw.

Takotsubo cardiomyopathy presents very similar to acute coronary syndrome and can initially be difficult to diagnose [7]. Some potential, but infrequent complications of the disease include hypotension, ventricular rupture, thrombosis involving the LV apex, and torsades de pointes. Some causes of the disease may include coronary spasm, coronary microvascular dysfunction, catecholamine toxicity, and myocarditis. The pathophysiology of Takotsubo, however, is not fully understood [7]. Currently, catecholamine cardiotoxicity and microvascular dysfunction are the most supported theories (Figure 1).

Takotsubo has become more recognized worldwide, but it is still frequently misdiagnosed [8]. The diagnosis of Takotsubo includes a normal epicardial coronary artery and an acute balloon-like dilation in the LV apex [1,2,7]. Per the Mayo Clinic diagnostic criteria for Takotsubo cardiomyopathy, there must also be an absence of recent significant head injury, myocarditis, or hypertrophic cardiomyopathy [7].

Treatment of Takotsubo cardiomyopathy is mainly symptomatic treatment in the acute phase [7]. Treatment with a beta-blocker or alpha-adrenoceptor agonist should be considered in patients with severe LV outflow tract obstruction and hemodynamic instability. Calcium channel blockers can also decrease LV outflow tract pressure gradient and help with vasospasms. It is important to avoid treatment with nitrites or inotropic drugs [7,9]. Patients with loss of motion of the LV apex should be considered for anticoagulation therapy to reduce the risk of thromboembolism [7]. Patients that are hemodynamically stable are often treated with beta-blockers, angiotensin-converting enzyme inhibitors, and diuretics. However, there is some conflicting evidence that chronic treatment with these medications may not provide any benefit in patients with Takotsubo cardiomyopathy [10].

## Conclusions

Takotsubo cardiomyopathy typically occurs in postmenopausal women and presents very similar to acute coronary syndrome. Despite the symptoms, EKG changes, and elevated cardiac enzymes, cardiac catheterization will reveal no significant atherolosclerotic coronary artery stenosis. Echocardiogram in patients will show depressed left ventricular function with cardiac wall motion abnormalities. Stressors, including emotional stress such as in this case, can lead to Takotsubo, which is why it has been given the name “broken heart syndrome”. To help differentiate acute coronary syndrome from Takotsubo, increased awareness of Takotsubo cardiomyopathy is important to realize that physical and emotional stressors can cause this disease.
